# Focus on international research strategy and teaching: the FIRST programme

**DOI:** 10.1007/s40037-013-0098-4

**Published:** 2013-12-05

**Authors:** Halah Ibrahim, Satish Chandrasekhar Nair

**Affiliations:** Academic Affairs Department, Tawam Hospital, PO Box 15258, Al Ain, Abu Dhabi, United Arab Emirates

**Keywords:** Research strategy, Research teaching, International medical education, Graduate medical education

## Abstract

Research, education and patient care are important missions of academic health centres and their respective teaching programmes. Yet, teaching hospitals struggle with ways to effectively support quality research initiatives. These issues are even more pronounced in countries with developing medical education systems where inconsistencies in training programme structure and quality, along with the lack of uniformity in the backgrounds and experience of the faculty, can hinder meaningful research activities. Hospital leaders recognized the need to inculcate both the desire and the ability to conduct quality research into every postgraduate training programme. Given the lack of global benchmarks, the institution developed and implemented the FIRST Programme (Focus on International Research Strategy and Teaching), a hospital-wide approach to research strategy training and support in 2010–2012. Over a 3-year period, the number of resident and faculty research activities has more than doubled. The types of research studies have also changed over the past few years, with an increase in basic science and randomized clinical trials. Our experience with implementing an institution-wide research initiative has been quite encouraging. Through leadership commitment, the institution has witnessed substantial increases in both trainee and faculty scholarship over a 3-year period.

## Background

Research, education and patient care are important missions of academic health centres and their respective teaching programmes. Studies have shown that resident participation in research is correlated with an increased number of lifetime publications [1], encourages future careers as academics [2], increases subspeciality fellowship training opportunities [3], and can ultimately lead to improved patient care [4]. Despite the importance of resident scholarship, graduate medical education has historically focused on clinical service and education, with patchy and inadequate attention to research training [5]. In recognition of the importance of incorporating research into residency training, regulatory bodies worldwide, including the Arab Board for Medical Subspecialities, the United States based Accreditation Council for Graduate Medical Education and the United Kingdom’s Royal College of General Practitioners require scholarly activity for both trainees and faculty. Strategies for improving research productivity have included recruiting a post-doctoral researcher to coordinate and support resident projects [6]; implementing a multimodal educational intervention to provide training in research and statistics [7]; and developing a formal, structured research rotation [8]. These approaches, almost exclusively at the individual programme level, have had varying degrees of success, and training programmes in all disciplines continue to struggle with ways to provide effective research experiences [9]. These issues are even more pronounced in countries with developing medical education systems where inconsistencies in training programme structure and quality, along with the lack of uniformity in the backgrounds and experience of the faculty, can hinder meaningful research activities.

The United Arab Emirates (UAE) is a small nation bordering the Persian Gulf. Over the past two decades, the country has become a political and economic leader in the Arab World and is a rapidly developing region for medical education and biomedical research [10]. The opening of the first medical school in 1984 paved the way for the development of residency training programmes in the mid-1990s. Subsequently, small-scale, independent research initiatives developed in some residencies. However, research strategy was never formally introduced into the curriculum for postgraduate medical programmes. Without central oversight, inconsistencies developed in the quality and effectiveness of research training in the individual programmes and began to increase over time.

Medical education leaders in our institution recognized the need to inculcate both the desire and the ability to conduct quality research into every residency programme. Given the lack of global benchmarks, the institution developed and implemented a hospital-wide approach to research strategy training and support, the FIRST Programme (Focus on International Research Strategy and Teaching), in 2010–2012 using established principles of change management. We hope that lessons learned from our experience will provide a foundation for research strategy and teaching in similar international teaching hospitals.

## Setting and change model

Tawam Hospital is a Joint Commission International-accredited 467-bed tertiary care centre in Abu Dhabi in the UAE. Tawam sponsors nine residency and fellowship programmes and serves as a training site for medical, nursing, and clinical pharmacology students from various institutions of higher education. The institution received ACGME-International accreditation in 2012.

To bring about significant and enduring changes in the institution’s approach to research training, educational leaders turned to classic theories of organizational development and change management [11, 12]. The basic tenets include creating compelling reasons to change (unfreeze); implementing the necessary changes (move); and developing systems for sustainability (refreeze) [12].

## Programme development

Prior to 2010, few residents and faculty at Tawam Hospital were involved in research. Review of 2008 and 2009 submissions to the Institutional Review Board (IRB) did not reveal any resident collaborators. Peer-reviewed publications were infrequent for faculty and consisted mainly of case reports. Graduate surveys revealed that only 47.1 % of graduating residents considered themselves competent to evaluate clinical evidence and even fewer (35.3 %) felt comfortable conducting clinical research. The most commonly identified barriers included lack of time due to competing pressures of patient care and education, lack of knowledge of basic research methodology and statistics, and unavailability of knowledgeable mentors.

### Unfreeze

A major component of FIRST is communication between hospital administration and faculty. The pursuit of excellence in research was added to the institution’s medical education vision statement, thereby, highlighting the importance of research as a core component of medical training. Faculty development sessions, which previously focused on teaching and evaluation skills, included workshops on basic statistics and research methodology. The Director of Clinical Research, a PhD with significant experience in conducting clinical trials, took a lead role in implementing the institution’s strategy for research training. Hospital-wide Continuing Medical Education (CME) lectures were conducted to create awareness. Topics included descriptions of the basic types of research, introduction to the IRB, and formulating a scientific hypothesis.

### Move (transition)

Barriers to conducting effective research were addressed during this stage. The institution’s main objectives were to: (1) provide research methodology training, (2) allocate protected time for scholarly activity, and (3) identify and train mentors.

1. *Research methodology training* The Research Director conducted a series of workshops on research methodology. The timing of the lectures concurred with resident protected academic time, leading to an over 80 % attendance rate. Topics included opportunities and challenges for clinical trials, research methodology, and research ethics. In addition to the hospital-wide sessions, workshops were scheduled with the individual training programmes, covering topics such as study designs, basic statistical concepts, and critical appraisal of the medical literature. Journal Club sessions were started in all the training programmes to provide the trainees with an opportunity to utilize the information acquired during the workshops.
2. *Allocating protected time for scholarly activity* The commitment to research was further demonstrated in the individual training programmes by allocating 2–4 week formal research blocks annually. Ongoing projects were continued during all 3–5 years of residency. The curriculum of the research block varied by discipline, but objectives included identifying a faculty mentor, designing a research study, performing a literature review and submitting an application to the local IRB. In most programmes, research rotations replaced electives so as not to disrupt clinical services.
3. *Identifying and training mentors* A 3-day workshop on research methodology, conducted by external experts, was offered to all programme directors and core teaching faculty. All workshop participants agreed to serve as faculty mentors for resident research projects. On-going guidance and support was provided by the Research Director.

### Refreeze

To ensure that the commitment to support and mentor scholarly activity was upheld by the faculty, evaluation of their contribution to medical education was included as part of their annual performance appraisals. For trainees, participation in scholarly activity became an important component of evaluations and promotions. Resident abstract and poster presentations at national conferences were openly recognized and financial support was provided for travel. To further encourage research productivity, the number of peer-reviewed publications was introduced as a key performance indicator (KPI) for all Abu Dhabi teaching hospitals in 2010.

## Outcome

**Fig. 1:**
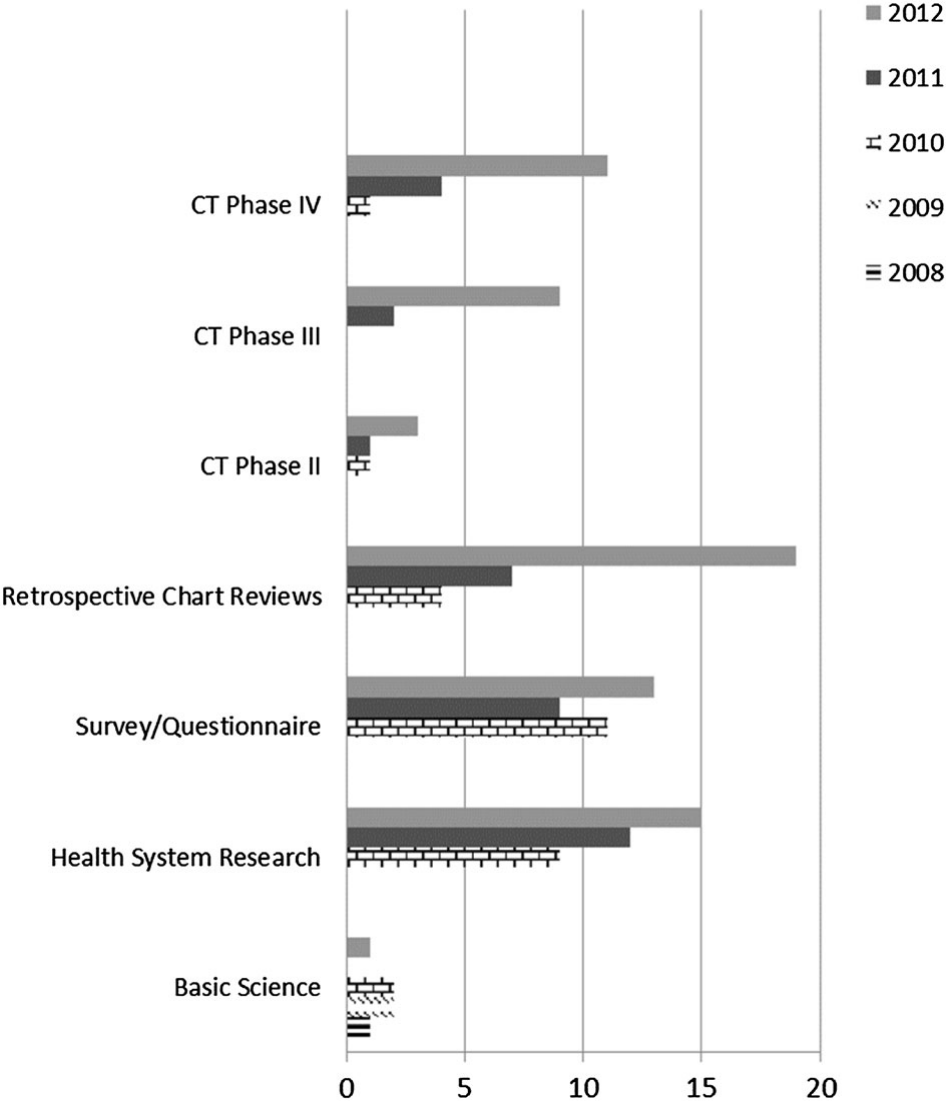
Number of research studies approved by IRB

Over a 3-year period (2010–2012), sessions dedicated to assisting residents with activities, such as idea generation and research design, increased by 733 %, including an increase in workshops focusing on methodology (345 %) and research ethics (183 %). A sevenfold increase from 2 resident projects in 2010 to 14 in 2012 was observed for research studies registered at the hospital’s central clinical research database and approved by the IRB. The institution also noted a transformation from limited basic science research conducted by the faculty prior to 2010 to translational studies after 2010, with many involving resident collaborators. There was a steady increase in retrospective chart reviews to assess incidence and prevalence, behavioural studies to assess quality of life, observational studies, such as disease registries, and phase II, III and IV clinical trials (Fig. 1). Phase 1 clinical trials are not permitted by the regulatory authorities in the UAE.

## Discussion

There are numerous obstacles to faculty and trainee involvement in research, including lack of protected time, inadequate mentorship opportunities and insufficient knowledge of research strategies. In addition to these challenges, international training programmes often lack the infrastructure and support to effectively encourage resident scholarly activity. Our experience with implementing an institution-wide research initiative has been quite encouraging. Through leadership commitment and the development of systems to embed (or refreeze) the new attitudes and behaviours into organizational culture, the hospital has witnessed substantial increases in both trainee and faculty scholarship over a 3-year period.

The current state of scholarly activity in our institution appears to be robust. However, funding, curricular changes, protected time and mentors are all necessary to support resident scholarship, and it is unclear how much effort must be expended to maintain this degree of research productivity, or if the manpower and financial resources have been diverted from other critical components of postgraduate training. Long-term sustainability of the programme and generalizability to other emerging health care systems will require better strategies for funding and innovative ways to improve education without escalating costs. Institutional collaborations and sharing of local resources and expertise are essential. International collaborations through teleconferencing of workshops and e-mentoring can also provide support with minimal increased costs. Through the support of academic and executive leadership, the FIRST Programme can be effectively implemented at other international teaching hospitals.

## Essentials

- Resident participation in meaningful research activity benefits the individual trainee and the hospital as a whole.
- Training programmes worldwide face barriers in supporting resident research productivity, including constraints in time, research methodology training and mentorship opportunities.
- An institution-wide approach to research teaching, if supported by hospital leadership, can successfully improve trainee and faculty research productivity.
